# Progress and Remaining Gaps in the Early Detection and Treatment of Breast Cancer

**DOI:** 10.3390/curroncol30030242

**Published:** 2023-03-08

**Authors:** Jean M. Seely

**Affiliations:** Department of Radiology, The Ottawa Hospital, University of Ottawa, Ottawa, ON K1N 6N5, Canada; jeseely@toh.ca

## 1. Introduction

Breast cancer affects too many of us. The preventable loss of life of mothers, sisters, daughters, grandmothers, fathers and beloved friends must be addressed through science. Female breast cancer has now surpassed lung cancer as the most diagnosed cancer in the world [1]. Almost 2.3 million new cases were diagnosed in women in 2020 [1]. Survival from breast cancer has steadily increased, and in Canada, breast cancer mortality has decreased by 46% since its peak in 1986, where the age-standardized mortality rate fell from 42.7 deaths per 100,000 to a projected rate of 23.1 deaths per 100,000 in 2021 [2]. While 65% of women with breast cancer are diagnosed at early stage I or stage II (localized), there are still far too many women who present at a stage III (regional) (27%) or stage IV (de novo metastatic disease) (6%) [3]. These proportions vary according to race and ethnicity [3]. Unfortunately, the early-stage diagnosis of breast cancer still progresses to metastatic breast cancer in 20–30% of women [4]. Improved survival has been achieved through the early diagnosis of breast cancer with screening and more effective and targeted treatments. Significant gaps remain, however, where an early diagnosis of breast cancer in certain populations is not achieved, including women with dense breasts, those of Black, Asian, Indigenous, and Hispanic ethnicities, and in women aged 40–49 years who are not routinely included in screening mammography programs. In addition, women at high risk who are 30 years and older and men may not be detected at an early stage. In this Special Issue of *Current Oncology on Breast Cancer Imaging and Therapy*, the latest evidence is presented on the early detection of breast cancer and treatment of breast cancer, highlighting the areas where this may be improved.

## 2. Adjuvant Endocrine Therapy for Breast Cancer

Rosso et al. evaluated the importance of adjuvant endocrine therapy for hormone-receptor-positive breast cancer in a retrospective observational study of 373 women with breast cancer [5]. In this survey study of 64% postmenopausal women, 84% experienced side effects, the most common being arthralgia, hot flushes and vaginal dryness. Significantly higher rates of side effects were found among women who also received adjuvant chemotherapy compared with those who did not (84.8% vs. 78.6%; *p* < 0.001). Premenopausal women were also more likely to experience side effects than postmenopausal women (92% vs. 75%; *p* < 0.001). In their study, 12% of patients stopped the adjuvant endocrine therapy mostly due to the side effects; those who did discontinue treatment more often reported severe side effects compared to those who did not (44% vs. 15%; *p* < 0.0001).

## 3. Men and Breast Cancer

Appiah et al. reported a prospective study of breast cancer among men in the United States; the research investigated 5216 men with breast cancer aged ≥40 years from the Surveillance, Epidemiology, and End Results program from 2000–2019, and studied the relation between breast cancer treatment and cardiovascular disease mortality [6]. They investigated the impact of race/ethnicity. After a median follow-up of 5.6 years, 37% (1914) of deaths occurred, of which 25% were attributable to cardiovascular disease and 35% to breast cancer. In multivariable analysis, men who received chemotherapy had a significantly elevated risk for cardiovascular-related death (HR: 1.55, 95% CI: 1.18–2.04). There was a significant interaction between race and ethnicity and cancer treatment on the risk of cardiovascular-disease-related mortality (*p* = 0.005), with higher levels among Hispanic (HR: 3.96, 95% CI: 1.31–12.02) than Black and White men.

## 4. High Risk and Breast Tissue Density

Rusnak et al. performed a prospective study of 139 women who were 40–69 years of age with a strong family history of breast cancer and no genetic mutations and were referred for high-risk assessment in a population-based breast cancer screening program [7]. They evaluated the impact of incorporating breast tissue density into risk assessment and found that 5.8% women had never had a screening mammogram. Of those who had mammography, the eligibility of 16.8% (22/131) was affected by their breast tissue density; 7% women with dense breasts became eligible while 10% with non-dense tissue became ineligible. The incorporation of density into risk stratification allowed for improved access to supplemental screening with breast MRI in women with dense breast tissue.

## 5. Stage of Breast Cancer and Screening

Wilkinson et al. evaluated the stage of breast cancer at diagnosis in a study of 55,490 women aged 40–59 years in Canada from 2010 to 2017 [8]. Using the Canadian Cancer Registry, the authors found marked differences in the stages of breast cancer in women aged 40–49 years compared with those aged 50–59 years; there were significantly lower proportions of stage I BC (35.7 vs. 45.3%; *p* < 0.001), and greater proportions of stage II (42.6 vs. 36.7%, *p* < 0.001) and stage III (17.3 vs. 13.1%, *p* < 0.001) in women in their 40s compared with the 50s. The authors evaluated the impact of organised screening programs on the stage of breast cancer at diagnosis. Jurisdictions that included women in their 40s in population-based screening programs had higher proportions of stage I (39.9% vs. 33.3%, *p* < 0.001) and lower proportions of stages II (40.7% vs. 43.7%, *p* < 0.001), III (15.6% vs. 18.3%, *p* < 0.001) and IV (3.9 vs. 4.6%, *p* = 0.001) in women 40–49 years old compared with their peers in the urisdictions that did not include them. A downstream impact was also seen in women in the 50s where screening practices for women aged 40–49 affected women aged 50–59 years; jurisdictions that did not screen women in their 40s had higher proportions of stage II (37.2% vs. 36.0%, *p* = 0.003) and stage III (13.6% vs. 12.3%, *p* < 0.001) in women aged 50–59 years as compared with programs that included the women in their 40s.

## 6. Knowledge about Screening and the Overdetection of Breast Cancer

Alenezi et al. performed a cross-sectional study in Saudi Arabia among 414 randomly selected female healthcare workers to assess their level of knowledge, attitude to breast cancer and barriers to mammography screening [9]. A high rate of a lack of knowledge was found, with 48.6% of the health care workers having a very low knowledge of breast cancer, and there was a significant negative correlation between a lack of knowledge and barriers to screening. The most important barriers related to screening included apprehension about radiation exposure (57%), fear of pain related to the mammographic examination (55.8%), fear of discovering breast cancer (57.2%) and fear of not knowing the procedure (48%). Logistic regression analysis found that physicians (*p* < 0.016) and workers older than 30 years of age (*p* < 0.03) were significantly more likely to have higher awareness about mammograms. This information may help target educational programs to improve mammography screening.

One of the most cited harms of breast cancer screening is that caused by the overdiagnosis or the overdetection of a breast cancer that would not have otherwise been found without screening, and that would not have led to the harm or death of the woman. Yaffe and Mainprize [10], in an excellent review, explore the phenomenon of overdetection, the methods for accurate estimation and the reasons for the variability in published estimates, including the very high value used by the Canadian Breast Cancer Screening Studies (CNBSS) [11] that inform the Canadian Task Force on Preventive Health Guidelines for Breast Cancer Screening. They demonstrate unequivocally that in situ carcinomas are the most common cause of overdetection and should not be overtreated, and that overdetection is a far greater problem in older than younger women due to more competing causes of death in the older ages.

## 7. Contrast-Enhanced Mammography

The ability of contrast to detect cancers in dense breast tissue on mammograms is now well established with contrast-enhanced breast MRI and contrast-enhanced mammography (CEM). However, it is well recognized that many benign breast lesions will also enhance with contrast. In this special Issue, Fusco et al. performed a study to discriminate between benign and malignant breast lesions with radiomic metrics extracted from CEM and DCE-MRI images [12]. A total of 79 pathologically proven breast lesions (48 malignant and 31 benign lesions) in 54 patients were studied. Various features on both modalities were studied, with the two best predictors found with an Area Under the Curve (AUC) of 0.71 on the mediolateral oblique (MLO) image of CEM. When all 18 features derived from MRI and CEM were combined, the AUC reached 0.88. The use of morphological assessment was insufficient while the radiomic features allowed for a better discrimination of benign- from malignant-enhancing breast lesions. In another study of CEM, Steinhof-Radwanska et al. [13] studied its use on patients with breast cancer treated with neoadjuvant chemotherapy. In their retrospective study of 63 patients with breast cancer who underwent CEM to assess their chemotherapy response, they found that CEM was highly sensitive in detecting a complete response to chemotherapy (85.7%), but it tended to underestimate the correct tumor dimensions. Recognizing this morphological limitation, CEM is a viable alternative to contrast-enhanced MRIand is effective in the detection of a complete response to chemotherapy.

## 8. Dense Breast Tissue

In a comprehensive review, Gordon [14] summarizes the impact of dense breasts and the stage of breast cancer at diagnosis. In it, she cites the relative loss of breast cancer mortality reduction in screened women with dense compared with non-dense breasts (13% vs. 41%), and the increased breast cancer mortality relative risk of 1.91 in women with dense breasts. The various supplemental screening modalities are discussed, including those with the greatest ability to reduce the inequity of screening for breast cancer in dense breasts, and the balance of risks and benefits of each one.

In the study by Hadadi et al. [15] of 534 Australian women recalled from screening with mammographic abnormalities and subsequent digital breast tomosynthesis (DBT) and breast ultrasound, breast tissue density was found to correlate significantly with recall rates. Mammographic abnormalities were more likely to be recalled in women with dense breasts. Breast ultrasound was shown in this study to be more useful than DBT at reducing the rate of unnecessary breast benign biopsies.

## 9. Harms of Not Screening

The harms and benefits of screening mammography are not fully understood by many oncologists and physicians. World expert, Harvard Professor Kopans review, named, “Misinformation and Facts about Breast Cancer Screening”, listed the 10 reasons that screening mammography is effective at saving lives from breast cancer by 40% or more [16]. Citing widely from the literature, he uncovers the myths that prevent women in their 40s from routinely being included in screening mammography programs. He summarizes the 1980 Canadian Breast Cancer Screening Studies’ (CNBSS) significant flaws that include the subversion of the randomization allocation, poor image quality of the mammography, the inclusion of many symptomatic women and allocation of more to the so-called “screening” arm, and shows why the CNBSS are not credible to determine screening policies.

A review by Appavoo illustrates the influence of the CNBSS on national and international breast cancer screening guidelines [17]. She provides insight into how the flawed studies came to be included in reviews that inform guideline processes. Illustrating the lack of expertise in healthcare guideline processes and drawing on examples of epistemic trespassing, manufactured doubt, and the misuse of the evidence-based review principles, she lists many reasons for the ongoing inclusion of CNBSS in the body of mammography screening evidence. She suggests reforms for the creation of new breast cancer screening guidelines that include expert knowledge and sensitivity to context and the need for fundamental change.

In the last but potentially most clinically relevant article, Dale, Tomaso and Gay summarize the lived experiences of many patients with breast cancer, many of whom were impacted by outdated screening practices [18]. Told through eight stories of women personally affected by breast cancer, the authors depict variable screening practices across Canada for women in their 40s, contrasting the early stage of breast cancer in two different 40 year old women who were both detected by screening mammography in the provinces of British Columbia and Prince Edward island, while another woman in Alberta presented with stage IV breast cancer two years after being dismissed by her family physician when she requested to be screened with mammography, citing guidelines that she was too young. The healthcare costs and emotional and physical toll on women diagnosed at a later stage highlight the harms of not screening women for breast cancer. The authors call on the medical community to do everything possible to reduce advanced stage breast cancers by harmonizing and optimizing screening practices.

## 10. Conclusions

Breast cancer is the most frequently diagnosed cancer in women. However, information about breast cancer screening is still limited and affects our ability to diagnose breast cancer at an early stage. Knowledge and strong evidence are essential to provide the rationale for the early detection of breast cancer, and this Special Issue aims to highlight ways to further improve survival and quality of life in women diagnosed with breast cancer. As a physician and breast radiologist who sees the harm of advanced breast cancers at diagnosis, I dream of the ways that we can reduce the rate of advanced breast cancer. Marie Curie said, “I was taught that the way of progress was neither swift nor easy.” As illustrated in this Special Issue, with improved screening practices that incorporate breast tissue density, we are making progress. Let us keep this in the realm of the possible and minimize the harm of breast cancer to our patients.
